# Primary Malignant Melanoma of the Lung (PMML); A Case Report

**DOI:** 10.7759/cureus.36850

**Published:** 2023-03-29

**Authors:** Pradeep Kumar Mada, Meagan Garibay, Robbie L Graham

**Affiliations:** 1 Infectious Diseases, Comanche County Memorial Hospital, Lawton, USA; 2 Infectious Disease, Comanche County Memorial Hospital, Lawton, USA; 3 Pathology, Comanche County Memorial Hospital, Lawton, USA

**Keywords:** retroperitoneal lymphadenopathy, malignant melanoma, adrenal metastases, brain metastases，lung cancer, primary malignant melanoma of lung

## Abstract

Primary pulmonary malignant melanoma (also called primary malignant melanoma of the lung, or PMML) is an exceedingly rare non-epithelial neoplasm, accounting for 0.01% of all primary lung cancers. We report a case of a 63­-year-­old male with no comorbidities who was found to have a large right lung upper lobe mass and was diagnosed with metastatic primary malignant melanoma of the lung. The outcome for primary pulmonary malignant melanoma is grim, with 5-year survival less than 20%, but many patients have rapid progression and a short life span, even with intervention.

## Introduction

Malignant melanoma accounts for approximately 160,000 new cancer cases yearly and causes about 41,000 deaths annually [1]. Melanoma primarily occurs on the skin due to exposure to ultraviolet rays. However, it may also occur in other mucosal sites such as the paranasal sinuses, oral cavity, esophagus, larynx, vagina, anorectal region, and liver. Primary pulmonary malignant melanoma (also called primary malignant melanoma of the lung, PMML) is an exceedingly rare non-epithelial neoplasm, accounting for 0.01% of all primary lung cancers. A 2017 literature review found only 40 cases reported in English literature [2]. The mean age at diagnosis was 59 years old, and a slight male prevalence among the cases (21 male to 19 female). Slightly less than half (47.5%) of the cases experienced metastatic disease, and 65% experienced death within 18 months of diagnosis.

## Case presentation

**Figure 1:**
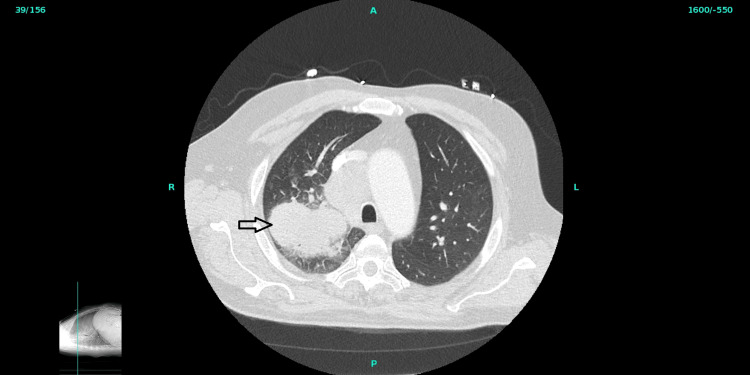
CT chest showing large right upper lobe lung mass

A 63­-year-­old Caucasian male with no comorbidities presented to the hospital with generalized weakness. In the emergency department (ED), the patient had a heart rate of 93 beats per minute, oxygen saturation of 97% on room air, and a temperature of 101.2F. Initial serum lab findings are shown in Tables 1-2. He complained of generalized weakness and denied having hit his head, head or neck pain, chest pain, shortness of breath, abdominal pain, blood in sputum, diarrhea, constipation, burning urination, blood in urine, change in vision, lightheadedness or any skin lesions or rash. He reported many years of tobacco chewing but denied alcohol, recreational drug use, skin or eye surgeries, and a family history of cancer. On physical exam, no skin and mucosal lesions or rashes were found. A single-view chest X-ray revealed a right hilar mass with a suggestion of right upper lobe post-obstructive atelectasis highly suspicious for malignancy. A computerized tomography (CT) scan of the chest showed a large right hilar mass contiguous with an ipsilateral supra hilar soft tissue component extending into the right upper lobe along the pleura surface measuring up to 5 cm in anteroposterior, 5.5 cm transverse and 13 cm in length (figure 1) encasing and narrowing the right upper and lower lobe pulmonary artery subsegmental branches. Interlobular septal thickening and adjacent ground glass opacities surrounding the right upper lobe lung mass may be secondary to compressive atelectasis, but lymphangitic metastasis cannot be excluded. The right upper and lower lobe pulmonary artery sub-segmental branches were narrowing without evidence of pulmonary embolus. There was no pleural or pericardial effusion. Subcarinal adenopathy measured up to 2.2 cm on the short axis; bilateral hilar adenopathy on the right measured up to 5 cm on the short axis, and on the left measured up to 2.5 cm on the short axis. Bulky para tracheal adenopathy measured up to 4 cm on the short axis. There was a 17 millimeter (mm) right hepatic lobe anterior segment parenchymal hypodensity and a 5.5 cm left adrenal gland metastasis. No pathological fractures were detected.

**Table 1:** A complete blood count (CBC) on admission

Parameter	Result	Reference range	Units
White blood cell	9.25	4.40-11.00	10x3/mm3
Red blood cell	4.36	4.50-5.90	10x6/mm3
Hemoglobin	11.7	13.2-16.5	grams (g) per deciliter (dL)
Hematocrit	37	39-49	%
Mean corpuscular volume	85	80-94	femtoliters (fL)
Mean corpuscular hemoglobin	27	27-31	picograms (pg)
Mean corpuscular hemoglobin concentration	32	33-37	g/dL
Red cell distribution width	13.3	11.5-16.1	%
Platelets	342	130-440	10x3/mm3
Mean platelet volume	10.3	7.2-11.1	fL
Neutrophil%	85	40-74	%
Lymphocyte%	7	19-48	%
Monocyte%	6.8	3.4-10.0	%
Eosinophil%	0.5	0.0-7.0	%
Basophil%	0.1	0.0-1.5	%
Neutrophil#	7.9	1.9-8.0	10x3
Lymphocyte#	0.7	1.0-4.9	10x3
Monocyte#	0.6	0.2-1.0	10x3
Eosinophil#	0.1	0.0-0.7	10x3
Basophil#	0.0	0.0-0.2	10x3

**Table 2:** A Comprehensive Metabolic Panel (CMP) on admission

Parameter	Result	Reference range	Units
Sodium	133	135-145	millimoles per litre (mmol/L)
Potassium	4.3	3.5-5.0	mmol/L
Chloride	95	96-110	mmol/L
Bicarbonate	23	21-31	mmol/L
Glucose	97	80-100	mg/dL
Blood Urea Nitrogen	22	6-21	mg/dL
Creatinine	1.29	0.6-1.4	mg/dL
Calcium	9.4	8.8-11.1	mg/dL
Total Protein	7.7	5.9-8.4	g/dL
Albumin	4.1	3.2-5.2	g/dL
Bilirubin Total	0.54	0.00-1.20	mg/dL
Alkaline Phosphatase	79	41-133	units per liter (u/L)
Aspartate Aminotransferase	15	7-39	u/L
Alanine Aminotransferase	9	2-54	u/L

**Figure 2:**
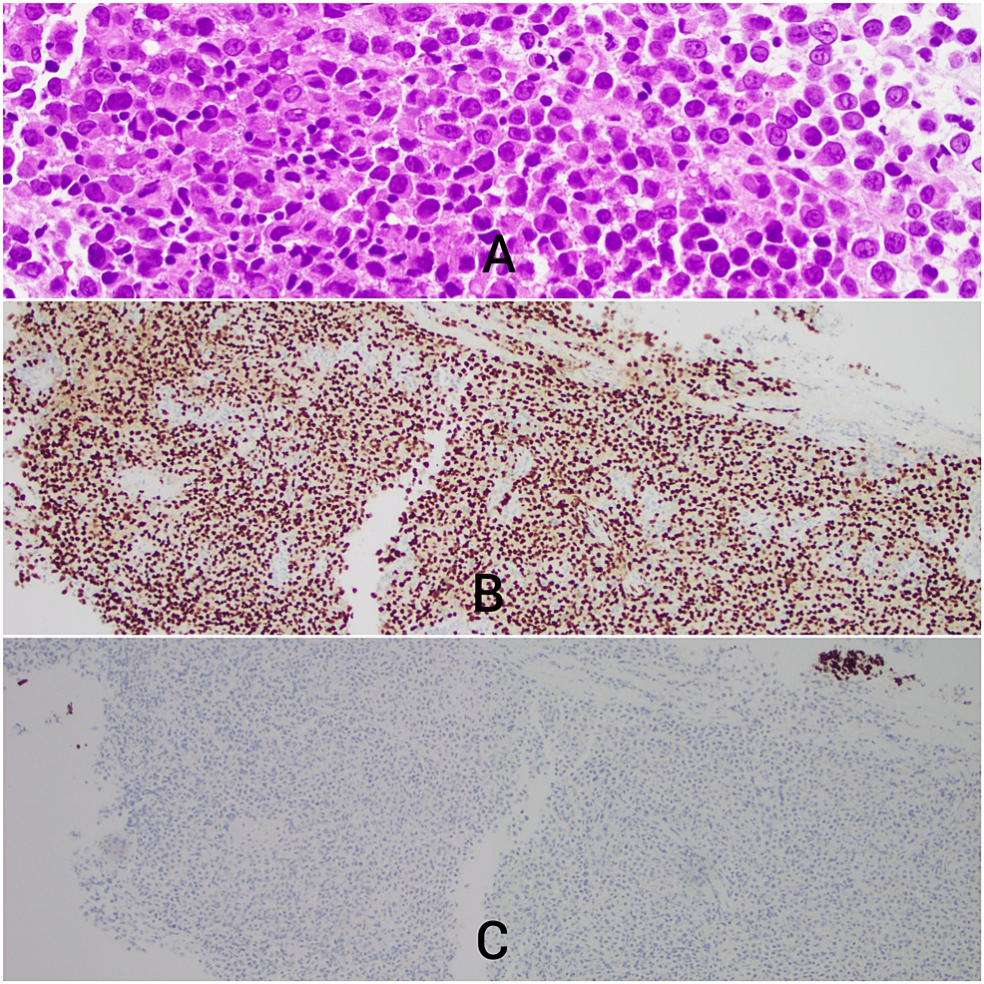
Lung biopsy. (A) Melanoma cells histology (original magnification 200x). (B) Melanoma-positive immunostaining with Sox-10 (original magnification 100x). (C) Melanoma negative immunostaining for Pan-CK ruling out carcinoma (original magnification 100x). Endoscopic biopsies of the right hilar mass were performed and submitted to Pathology for examination. Sections of these biopsies show sheets of atypical malignant cells (Figure A).  These cells are confirmed to be melanoma with positivity for Sox-10 immunohistochemistry (Figure B), and they are negative for pan-cytokeratin ruling out carcinoma (Figure C).

The carina was well visualized during bronchoalveolar lavage (BAL) and appeared sharp. The right lower lobe and middle lobe bronchi were clear on the right side. An obstructive lesion was present in the right upper lobe. It appeared friable and involved the origin of 2 of the 3 segments. That area was lavaged and collected in the Lukens tube. The pieces of the endo bronchial lesion were extracted. A right upper lobe needle biopsy revealed malignant melanoma (Figure 2).

**Figure 3:**
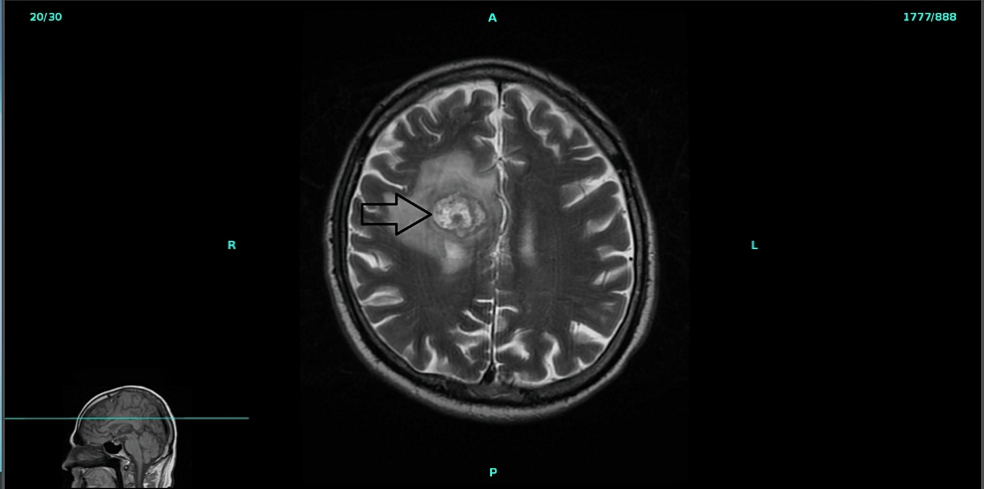
MRI brain showing a 3.4 cm ring-enhancing lesion in the medial aspect of the right parietal lobe.

**Figure 4:**
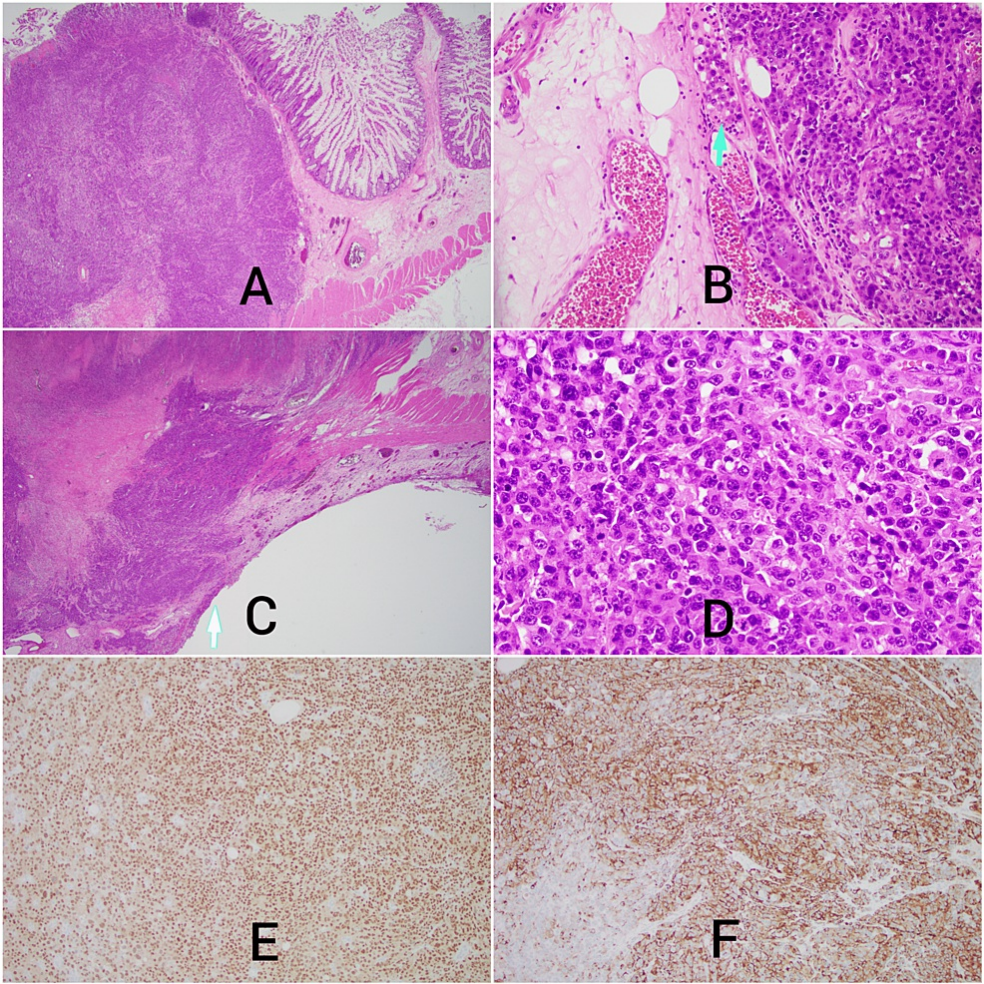
Small bowel resection. (A) Melanoma invading the small bowel (original magnification 20x). (B) Melanoma within lymphovascular spaces (original magnification 200x). (C) Melanoma perforates the small bowel with serosal involvement (original magnification 200x). (D) Melanoma cells histology (original magnification 400x). (E) Melanoma-positive immunostaining with Sox-10 (original magnification 100x). (F) Melanoma-positive immunostaining with HMB-45 (original magnification 100x). A 56 cm segment of the small intestine was subsequently excised and submitted to Pathology for gross and microscopic examination. Three separate masses were identified in the intestinal wall measuring 2.0 cm, 4.1 cm, and 4.5 cm in greatest dimensions. One of these masses was associated with a 3.7 cm perforation. Histologic sections of the masses showed similar findings with invasive melanoma involving the full intestinal wall thickness (Figure A). There was lymphovascular invasion (Figure B), and the tumor was perforated with serosal involvement (Figure C). A high-powered microscopic view shows the melanoma cells to be anaplastic with prominent nucleoli, moderate amounts of cytoplasm, and brisk mitotic activity (Figure D). Immunohistochemical stains further confirm the tumor to be metastatic melanoma with positivity for Sox10 (Figure E) and HMB-45 (Figure F).

Further imaging was done for staging. Brain magnetic resonance imaging (MRI) with and without contrast revealed a 3.4 cm ring ­enhancing lesion in the medial aspect of the right parietal lobe (Figure 3) and a more homogeneously enhancing 2.7 cm lesion in the inferior left cerebellar hemisphere consistent with metastatic melanoma. Fundoscopy did not reveal any ocular lesions. CT abdomen and pelvis with contrast revealed multiple lesions in the retroperitoneum, mesentery, left adrenal gland, and right gluteal soft tissues suspicious for metastasis. Oncology was consulted for further management of metastatic primary malignant melanoma. Radiation oncology was also consulted for brain metastases. His Eastern Cooperative Oncology Group (ECOG) Performance score was 3. He was started on dexamethasone 4mg every 6 hours. The patient was discharged in stable condition. He was treated with 27 Gray stereotactic radiation treatments in 3 fractions for his right parietal lesion. He left cerebellar lesions and was started on palliative immunotherapy with Nivolumab 1 mg/kg plus ipilimumab 3 mg/kg every 3 weeks for 4 cycles. He again presented after 2 weeks to the ED from the nursing home with three days of progressively worsening constipation, abdominal pain, and abdominal swelling. CT's abdomen pelvis demonstrated a small bowel obstruction with micro-perforation and numerous metastases. He underwent exploratory laparotomy with a small bowel resection of approximately 70 cm of distal ileum and primary stapled anastomosis. During surgery, a large metastatic implant in the distal ileum approximately ­20 cm from the ileocecal valve, a smaller implant approximately 15 cm proximal to the distal implant, and a small perforated bowel with frank contamination were found. The entire segment of the small bowel containing perforation and implants was resected and sent for pathology (Figure 4). After discharge from the hospital, due to the deterioration of his clinical condition, he was made to hospice care and passed away 2 months later.

## Discussion

Since there was no history of skin or eye surgeries, no skin and mucosal lesions were found on physical exam, and fundoscopy did not reveal any ocular lesions, it was diagnosed as PMML in our patient. Primary pulmonary malignant melanoma is typically endobronchial, and symptoms are usually correlated to the disruption of this area, with cough, hemoptysis, shortness of breath, pleuritic pain, pneumonia, lobar collapse, or atelectasis among common complaints that cause patients to seek medical attention [1]. Some patients may present with nonspecific or no complaints, and lesions may instead be discovered incidentally, like in our patient. If metastatic disease is already present, presenting symptoms may be correlated to tumor invasion in those areas, such as neurological deficits in a patient with brain metastases [3].

Endobronchial findings may be present; during a literature review of 13 patients diagnosed with primary pulmonary melanoma who underwent a diagnostic bronchoscopy, 8 had a detectable tumor during the exam [4]. Additionally, surgical or autopsy findings in 16 out of 20 patients found an endobronchial tumor spread.

Before diagnosing a patient with primary pulmonary malignant melanoma, all other possible sites of a primary melanoma should be ruled out. This includes diagnostic modalities such as a physical exam focusing on systematically exploring the patient's skin surface, including scalp; gastrointestinal endoscopy and colonoscopy; endoscopy of the nasal cavity and sinuses; gynecological examination in females; and positron emission tomographic (PET) scanning [3]. 

Pathophysiology

The exact pathogenesis of primary pulmonary malignant melanoma is unclear. There are several theories on how it occurs, the most popular two being the migration of melanocytes during embryonic development or the proliferation of melanocytes in the larynx and esophagus [1]. When no primary lesion can be identified, some studies have pointed to the possibility of primary malignant cutaneous melanocytes that form and disappear after metastasizing (5 to 10 percent of patients with metastatic melanoma have a primary melanoma of unknown origin) [3].

Allen and Drash proposed nine criteria for establishing a diagnosis of primary pulmonary malignant melanoma: (i) no history suggestive of a previous melanoma; (ii) no identifiable melanoma at any other site at the time of surgical intervention; (iii) a solitary tumor in the lung surgical specimen; (iv) tumor morphology consistent with that of a primary tumor; (v) no evidence at autopsy of a primary melanoma elsewhere; (vi) melanoma cells confirmed by immunohistological staining for S-100 and HMB-45; (vii) evidence of junctional change; (viii) nesting of cells beneath the bronchial epithelium; and (ix) invasion of the intact bronchial epithelium by melanoma cells [5]. Unlike other lung malignancies, PMML does not correlate with cigarette smoking [6].

Treatment

Surgical intervention to resect the tumor within an acceptable oncological margin, lobectomy, or pneumonectomy is the treatment of choice [1]. In cases where surgery is impossible or not desired by the patient, chemotherapy, radiation, and immunotherapy may be considered. The chemotherapy medication is dacarbazine, typically combined with immunotherapy such as interleukin-2 or interferon [3].

Unfortunately, the prognosis for primary pulmonary malignant melanoma is grim - five-year survival estimates may be at least 10%. However, many patients have rapid progression and short survival, even with the intervention [1,7].

## Conclusions

PMML is a rare, invasive neoplasm with a propensity toward recurrence and a poor prognosis; however, early detection and treatment can improve outcomes. The management includes an aggressive surgical approach, combined with or without chemoradiation or immunotherapy based on staging.
